# Effect of partial splenic embolization on transarterial chemoembolization for hepatocellular carcinoma with hypersplenism

**DOI:** 10.1097/MD.0000000000026441

**Published:** 2021-07-02

**Authors:** Jibing Liu, Zhijuan Wu, Jianxin Zhang, Yinfa Xie, Peng Sun, Huiyong Wu, Xu Chang, Lin Zhang, Fengyong Liu

**Affiliations:** a Department of Interventional Oncology, Shandong Cancer Hospital and Institute, Shandong First Medical University and Shandong Academy of Medical Sciences; b Department of Geriatrics, Jinan Central Hospital Affiliated to Shandong First Medical University, Jinan, Shandong; c Department of Intervention Therapy, General Hospital of the Chinese People's Liberation Army, Beijing, China.

**Keywords:** hepatocellular carcinoma, hypersplenism, liver function classification, peripheral blood, partial splenic embolization, transarterial chemoembolization

## Abstract

This study retrospectively studied transarterial chemoembolization (TACE) combined with partial splenic embolization (PSE) in the treatment of hepatocellular carcinoma (HCC) with severe hypersplenism.

Seventy patients with HCC in Barcelona Clinic Liver Cancer (BCLC) stage B or C with hypersplenism were divided into non-partial splenic embolization group (N-PSE, n = 51) and partial splenic embolization group (PSE, n = 19). The N-PSE group was further divided into N-PSE with mild to moderate hypersplenism (N-PSE-M, 47 cases) and N-PSE with severe hypersplenism (N-PSE-S, 4 cases).

In the PSE group, leukocytes, neutrophils, lymphocytes, and platelets were significantly increased (*P* < .05) and were significantly different from that in the N-PSE group (*P* < .05). In the N-PSE group, except for a slight increase in neutrophils, other blood cells were decreased, including lymphocytes that were significantly decreased (*P* < .05). There was no significant difference in the changes of liver function between the 2 groups before and after surgery (*P* > .05). The analysis showed a significant increase in ascites after 6 months of TACE in the N-PSE group (*P* < .05). According to the follow-up results, the median overall survival (OS) in the PSE group was 24.47 ± 3.68 (months) and progression-free survival (PFS) was 12.63 ± 4.98 (months). Regardless of OS or PFS, the PSE group was superior to the N-PSE group and its subgroups, with a statistically significant difference in PFS between the N-PSE group and PSE group (*P* < .05). Moreover, the time of extrahepatic progression was significantly earlier in the N-PSE group than in the PSE group (*P* < .05). N-PSE-S group had the worst prognosis, and PFS and OS were worse than the other 2 groups, suggesting that PSE in severe hypersplenism may improve PFS and OS.

In patients with HCC and severe hypersplenism, TACE should be actively combined with PSE treatment.

## 1 Introduction

Hepatocellular carcinoma (HCC) is the sixth most common tumor and the fourth leading cause of cancer mortality.^[1]^ Most HCCs are already in the middle and advanced stages when they are diagnosed, and transarterial chemoembolization (TACE) is the preferred or important treatment,^[2,3]^ which can significantly prolong the survival time of HCC patients. However, >85% of patients with HCC have cirrhosis.^[4]^ Hypersplenism caused by long-term liver cirrhosis tends to increase the risk of bleeding, infection, and liver function deterioration,^[5]^ and thus, is often considered as a significant factor limiting anticancer therapy.

Since Maddison first used partial splenic embolization (PSE) to treat hypersplenism in 1973,^[6]^ PSE has gradually replaced surgical resection as the primary treatment for hypersplenism being less invasive, more effective, and having the ability to preserve some of the splenic functions.^[7,8]^

The spleen is the largest immune organ in the body. It is still unclear how TACE combined with PSE affects the treatment of HCC in patients with HCC and hypersplenism. This article intends to retrospectively study the effect of PSE on the treatment of HCC with TACE and to explore the possible influence mechanisms.

## 2 Methods

### 2.1 Patients and dividing groups

**Figure 1:**
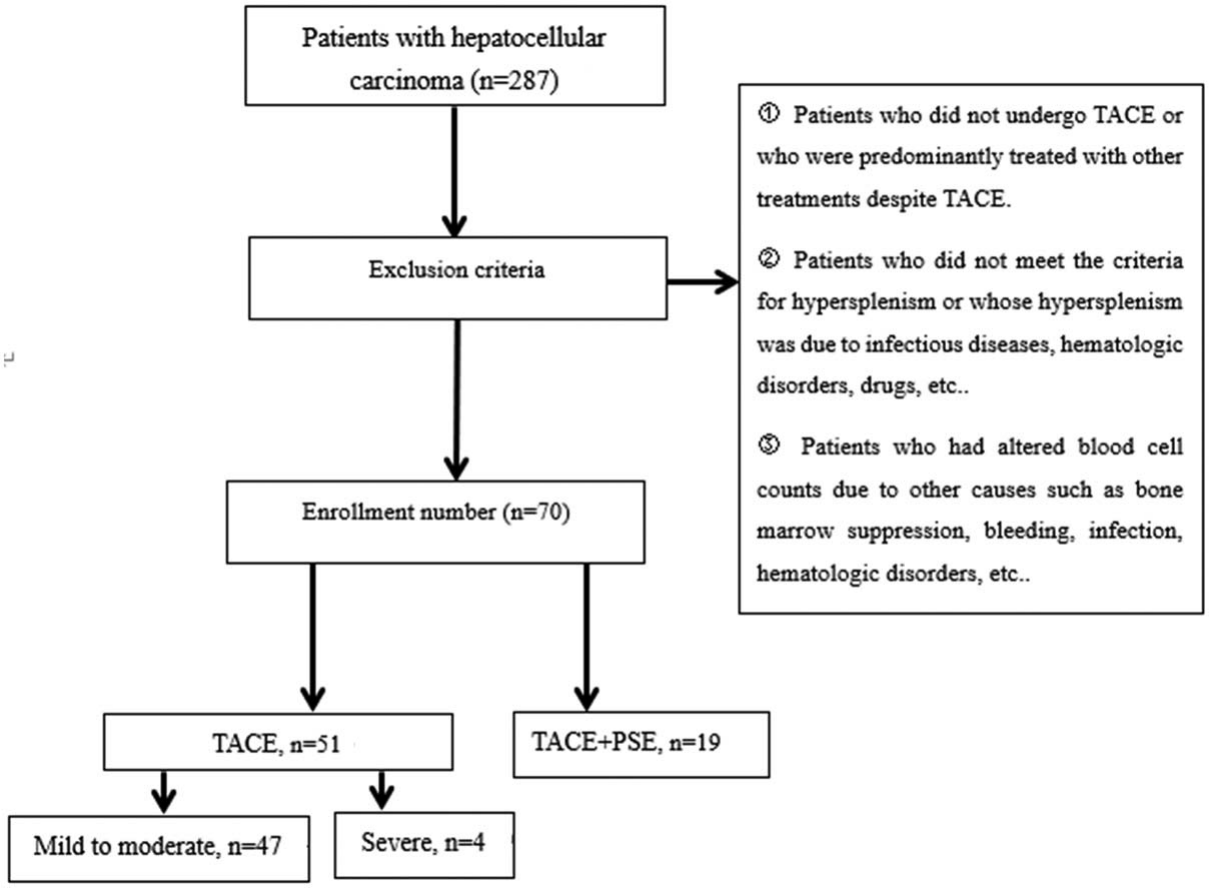
Flowchart of patient selection. Patient selection and grouping.

As shown in Fig. 1, a total of 287 patients with a pathological or clinical diagnosis of HCC were admitted to the second ward of the Interventional Department of Shandong Cancer Institute for Prevention and Treatment between July 1, 2015 and July 1, 2018. The enrolled patients were diagnosed with Barcelona Clinic Liver Cancer (BCLC) stage B or C, treated with TACE as primary treatment. They suffered splenomegaly caused by liver cirrhosis, had spleen index >492 cm^3^, underwent hemocytopenia, peripheral blood leukocyte count <4.0 × 10^9^/L, neutrophil count <2.0 × 10^9^/L, erythrocyte count <3.5 × 10^12^/L, hemoglobin (Hb) <110 g/L, platelet (PLT) count <100 × 10^9^/L, and Eastern Cooperative Oncology Group performance status ≤ 1. Exclusion criteria were: patients who did not undergo TACE or who were predominantly treated with other treatments despite TACE; patients who did not meet the criteria for splenomegaly or whose splenomegaly was due to infectious diseases, hematologic disorders, drugs, etc; patients who had altered blood cell count due to other causes such as bone marrow suppression, bleeding, infection, hematologic disorders, etc; patients who had an Eastern Cooperative Oncology Group ≥2.

**Figure 2:**
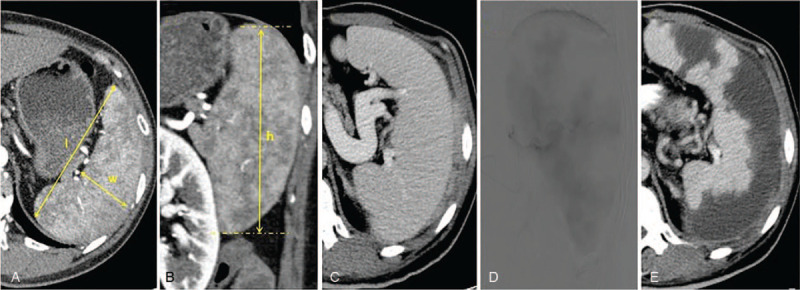
A patient with hypersplenism and the procedure of PSE. (A, B) Spleen index measurement method (*l* length, *w* width, *h* height). (C, D) PSE. PSE = partial splenic embolization.

The diagnostic criterion for computed tomography (CT) splenomegaly was established assessing the size of the spleen using the splenic index method. The specific method was to measure the following: length (*l*), which was the longest diameter between the 2 poles of the spleen in the transverse position; the width (*w*), which was the measurement between the medial and lateral margins of the spleen at the level where the longest diameter was located, perpendicular to the longitudinal line at the splenic portals; the height (*h*), which was the vertical distance from the upper to lower margins of the spleen. The splenic index was then calculated as follows: splenic index = *l* × *w* × *h* (Fig. 2A and B), with a splenic index >492 cm^3^ defined as splenomegaly.^[9]^

According to whether partial splenic artery embolization was performed, cases were divided into non-partial splenic embolization group (N-PSE) and partial splenic embolization group (PSE).

The N-PSE group was divided into 2 subgroups according to the degree of hypersplenism. Patients in the non-partial splenic embolization with mild to moderate hypersplenism group (N-PSE-M) had peripheral blood leukocyte counts of 2.0–4.0 × 1 0^9^/L, neutrophil counts of 1.0–2.0 × 10^9^/L, erythrocyte counts of 1.5–3.5 × 10^12^/L, Hb of 60–110 g/L and PLT count of 50–100 × 10^9^/L. Patients in the non-partial splenic embolization with severe hypersplenism group (N-PSE-S) had peripheral blood leukocyte counts <2.0 × 10^9^/L, neutrophil counts <1.0 × 10^9^/L, erythrocyte counts <1.5 × 10^12^/L, Hb <60 g/L, and PLT counts <50 × 10^9^/L.

### 2.2 TACE and PSE procedures

First, the patient was placed in the supine position. Then the local anesthesia was administered after routine surgical sterilization and placement of sterile drapes. According to the Seldinger technique, one side of the femoral artery was punctured to establish a vascular channel, and a 5Fr catheter (Terumo, Japan, or Cook, Indiana, USA) was placed under digital substraction angiography for hepatic angiography to show the tumor supplying artery. The 2.7Fr microcatheter (Progreat; Terumo, Japan) was superselected into the blood supply artery, and the angiography confirmed that the microcatheter was accurately positioned. Emulsifiers, such as epirubicin (Pfizer Pharmaceutical Co., Ltd., China), mitomycin (Jiangsu Hengrui Pharmaceutical Co., Ltd., China), and loplatin (Hainan Changan International Pharmaceutical Co., Ltd., China) with iodinated oil (Jiangsu Hengrui Medicine Co., Ltd., China, or Guerbet, France) were injected. Granular embolic agents, such as gelatin sponge pellets (Gelfoam, Hangzhou Alikang Medical Technology Co., Ltd., China) were injected until flow slowed or stagnated, with a re-imaging without tumor staining as the embolic endpoint. If there was obvious arteriovenous fistula, the embolization was adjusted. In addition to iodized oil embolization, larger diameter gelatin sponge particles or spring coils (Cook, USA) were used to embolize the fistula. The angiographic embolization image and preoperative CT were strictly compared with ensure that all tumors in the liver were embolized. If the embolization was not complete, the extrahepatic blood vessel was identified and embolized. The catheter was placed into the splenic artery, and splenic angiography was performed. An appropriate amount of gelatin sponge particles of approximately 1 to 2 mm in size were injected at the level of the splenic artery, with an embolic endpoint of approximately 50% to 70% of the angiographic embolic area (Fig. 2C–E).

After PSE surgery, oral antibiotics were routinely administered for 10 days to prevent infection. TACE treatment was repeated after 1 to 3 months according to the imaging, alpha-fetoprotein level, and patient's condition, and the patient with the stable condition was followed until the tumor progressed. According to the changes in the condition, other treatments, such as targeted therapy and radiotherapy, were added.

### 2.3 Follow-up

The upper abdominal enhanced CT scan, chest CT, liver function, and coagulation function were reviewed 1 to 3 months after TACE (±PSE). Other tests included brain CT review every 3 months and whole-body bone scan every 6 months to assess metastasis. With modified Response Evaluation Criteria in Solid Tumours as the evaluation standard, the target tumor and all tumors were evaluated. Complete response (CR) indicated that all target lesions disappeared during the arterial phase enhancement. Partial response (PR) indicated the comprehensive reduction of the diameter of the target lesion during the arterial phase enhancement ≥30%. Stable disease indicated that the reduction did not reach the PR or the increase did not reach the progressive disease (PD). PD indicated that the sum of the diameter of the target lesions during the arterial phase enhancement image increased ≥20% or new lesions appeared.

The blood routine, liver function, coagulation function, and imaging changes during TACE+PSE or only TACE due to the first hypersplenism, and 3 and 6 months after surgery were recorded to evaluate the influence of splenic embolism on peripheral blood cells and Child-pugh score. The patients were followed up for at least 18 months, and PFS and overall survival (OS) were evaluated.

### 2.4 Study endpoints

The OS was recorded using the patient's diagnosis of HCC as the starting time and the death of the patient as the end time. The diagnosis to disease progression or the time of death or censoring was recorded as progression-free survival (PFS). In the assessment of PFS, disease progression included intrahepatic in situ progression, intrahepatic neoplastic metastasis, and extrahepatic progression, respectively. The patients were followed up for at least 18 months. OS was taken as the primary endpoint and PFS as the secondary endpoint.

### 2.5 Statistical analysis

For the baseline characteristics analyses, categorical variables were presented as frequencies and percentages (n [%]). A Chi-square test was used to compare categorical variables. For blood cell and liver function, continuous variables are expressed as means ± standard deviation. Shapiro-Wilk test and Levene test were used to test normality and homogeneity of variance. Paired *t* test or Wilcoxon Signed-Rank Test compared preoperative and postoperative changes of TACE or PSE+TACE, and the comparisons between groups were made by independent sample *t* test or Mann–Whitney *U* test. Survival was estimated by the Kaplan–Meier method, and any differences in survival were evaluated with a stratified log-rank test. COX univariate analysis screened the potentially useful independent variables (*P* < .15). After the Linear Regression ruled out obvious collinearity, the independent variables were included in the COX multivariate analysis. All tests were 2-sided, and *P* < .05 was considered statistically significant. All statistical analyses were performed with SPSS v23.0 for Windows (IBM, USA). The histogram was made using Graphpad prism 5.

## 3 Results

### 3.1 Baseline characteristics

Among the 180 cases of BCLC stage B or C HCC, there were 149 cases of splenomegaly and 70 cases of hypersplenism. Among these, 51 cases were not treated with PSE, and 4 cases were diagnosed with severe hypersplenism. Also, 19 cases with severe hypersplenism underwent PSE. Among 70 cases of hypersplenism, 45.7% had 1 type of blood cell reduction, 47.1% had 2 types of blood cell reduction, and 7.1% had 3 types of blood cell reduction. The cases with thrombocytopenia were the most common, accounting for 72.9%. Leukopenia was observed in 65.7% of cases, and erythrocytopenia or decreased Hb was the least frequent, accounting for 22.9%. Among them, mild to moderate hypersplenism was dominated by one type of hemocytopenia (59.6%), while severe hypersplenism was dominated by 2 or 3 types of hemocytopenia (82.6%).

Most of the patients were middle-aged and men with a history of hepatitis. The main tumor was located in the right lobe, with pseudo capsule around it, combined with portal vein tumor thrombus and BCLC at stage C. In addition to TACE treatment, 20 cases underwent targeting, ablation, surgery, radiotherapy, and other treatment methods; there was no significant difference between the groups. Followed up for at least 18 months, 63 cases died, 5 cases survived, and 2 cases were lost to follow-up (details are shown in Table 1). Compared with TACE, TACE combined with PSE increased the incidence of abdominal pain and fever, all of which improved with symptomatic management and no serious complications.

**Table 1:** Baseline characteristics of the study population and the target lesions.

	N-PSE				
Items	Mild to moderate	Severe	PSE	Total	*P*
Count	47 (67.1%)	4 (5.7%)	19 (27.1%)	70	
Hemocytopenia					
One type	28 (40%)	2 (2.9%)	2 (2.9%)	32 (45.7%)	.000
Two types	19 (27.1%)	2 (2.9%)	12 (17.1%)	33 (47.1%)	
Three types	0	0	5 (7.1%)	5 (7.1%)	
Gender					
Male	42 (60.0%)	4 (5.7%)	18 (25.7%)	64 (91.4%)	.639
Female	5 (7.1%)	0	1 (1.4%)	6 (8.6%)	
Age, yr					
>60	18 (25.7%)	0	8 (11.4%)	26 (37.1%)	.274
<60	29 (41.4%)	4 (5.7%)	11 (15.7%)	44 (62.9%)	
AFP, ng/mL					
>400	27 (38.6%)	2 (2.9%)	10 (14.3%)	39 (55.7%)	.912
<400	20 (28.6%)	2 (2.9%)	9 (12.9%)	31 (44.3%)	
Hepatitis	42 (60.0%)	4 (5.7%)	17 (24.3%)	63 (90.0%)	.790
Child-Pugh	5.68 ± 1.07	6.00 ± 0.82	5.63 ± 1.01	5.69 ± 1.03	.500
TB, μmol/L	27.82 ± 36.11	35.98 ± 15.37	23.11 ± 13.55	27.01 ± 30.61	.716
Albumin, g/L	38.97 ± 5.44	39.30 ± 3.87	38.83 ± 5.70	38.95 ± 5.37	.987
PT, s	11.80 ± 1.33	12.15 ± 0.96	12.36 ± 1.43	11.97 ± 1.35	.305
Ascites	1.17 ± 0.38	1.25 ± 0.50	1.16 ± 0.38	1.17 ± 0.38	.907
HE	1.00 ± 0.00	1.00 ± 0.00	1.00 ± 0.00	1.00 ± 0.00	1.000
Location					
Left lobe	11 (15.7%)	0	3 (4.3%)	14 (20.0%)	.460
Right lobe	36 (51.4%)	4 (5.7%)	16 (22.9%)	56 (80.0%)	
Diameter, cm					
>10	20 (28.6%)	3 (4.3%)	7 (10.0%)	30 (42.9%)	.373
<10	27 (38.6%)	1 (1.4%)	12 (17.1%)	40 (57.1%)	
BCLC					
Stage C	39 (55.7%)	3 (4.3%)	13 (18.6%)	55 (78.6%)	.420
Stage B	8 (11.4%)	1 (1.4%)	6 (8.6%)	15 (21.4%)	
Pseudocapsule	38 (54.3%)	3 (4.3%)	14 (20.0%)	55 (78.6%)	.801
Portal vein tumor thrombus	30 (42.9%)	2 (2.9%)	9 (12.9%)	41 (58.6%)	.441
Arteriovenous fistula	8 (11.4%)	1 (1.4%)	5 (7.1%)	14 (20.0%)	.671
Other treatments	10 (14.3%)	1 (1.4%)	9 (12.9%)	20 (28.6%)	.103
Survival condition					
Death	43 (61.4%)	4 (5.7%)	16 (22.9%)	63 (90.0%)	.530
Lost	4 (5.7%)	0	7 (10.0%)	7 (10.0%)	

AFP = Alpha-fetoprotein, BCLC = Barcelona Clinic Liver Cancer, HE = hepatic encephalopathy, N-PSE = non-partial splenic embolization, PSE = partial splenic embolization, PT = prothrombin time, TB = total bilirubin. *P *<* *.05 is defined as a significant difference.

After PSE in patients with hypersplenism, the increase of leukocytes, neutrophils, lymphocytes, and PLTs was obvious (*P* < .05) and was significantly different from that in the N-PSE group (*P* < .05). This suggested that PSE could significantly increase the white blood cell (WBC) and PLT count, while there was not an obvious effect in improving anemia (*P* > .05). In the N-PSE group, except for a slight increase in neutrophils, other blood cells decreased, and lymphocytes significantly decreased (*P* < .05).

**Figure 3:**
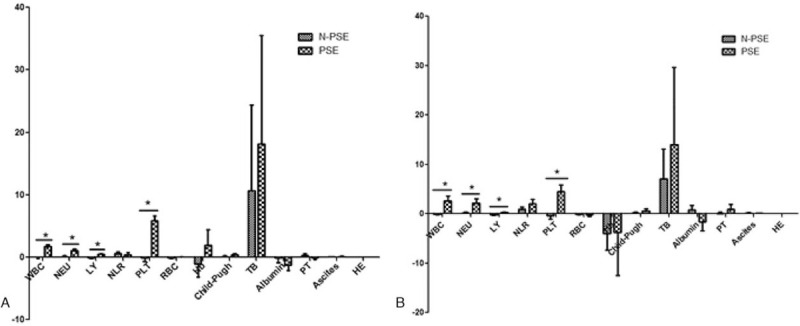
Histogram of the increase at 3 months and 6 months after the operation compared with the preoperative period. (A) Three months after operation compared with preoperative growth. (B) Six months after operation compared with preoperative growth. Note: PLT changed from ×10^9^/L to ×10^10^/L; ^∗^*P* < .05.

The liver function scores of the 2 groups had a tendency to increase, but there was no statistical significance (*P* > .05), and there was no significant difference in the changes of liver function between the 2 groups before and after surgery (*P* > .05). According to various analyses, ascites in the N-PSE group significantly increased 6 months after TACE (*P* < .05), suggesting that PSE may help slow down the increase in ascites (Tables 2–4, Fig. 3).

**Table 2:** Changes before and after PSE+TACE in PSE group (mean ± sd).

	Before operation	3 months after operation	3–0	*P*	6 months after operation	6–0	*P*
WBC (×10^9^/L)	2.67 ± 0.87	4.42 ± 1.16	1.75 ± 1.28	.000	5.23 ± 3.16	2.61 ± 3.47	.005
NEU (×10^9^/L)	1.56 ± 0.78	2.60 ± 0.89	1.04 ± 1.12	.001	3.56 ± 2.81	2.10 ± 3.18	.008
LY (×10^9^/L)	0.73 ± 0.28	1.19 ± 0.56	0.46 ± 0.47	.002	1.01 ± 0.58	0.22 ± 0.42	.094
NLR	2.41 ± 1.34	2.76 ± 1.74	0.35 ± 1.81	.409	4.18 ± 2.92	1.98 ± 3.13	.023
PLT (×10^9^/L)	55.79 ± 10.07	113.84 ± 45.00	58.05 ± 36.62	.000	104.17 ± 52.05	45.00 ± 44.80	.006
RBC (×10^12^/L)	3.94 ± 0.58	3.95 ± 0.62	0.01 ± 0.47	.958	3.95 ± 0.84	-0.24 ± 0.99	.308
Hb, g/L	123.11 ± 20.55	125.05 ± 17.54	1.95 ± 10.95	.485	123.92 ± 24.39	-3.83 ± 29.93	.844
Child-Pugh	5.63 ± 1.01	6.00 ± 1.20	0.37 ± 0.76	.052	5.67 ± 1.44	0.58 ± 1.44	.102
TB, μmol/L	23.11 ± 13.55	41.27 ± 78.74	18.16 ± 75.47	.327	33.28 ± 53.70	14.02 ± 54.02	.754
Albumin, g/L	38.83 ± 5.70	37.59 ± 6.00	-1.24 ± 3.71	.164	39.73 ± 4.83	-1.70 ± 5.95	.410
PT, s	12.36 ± 1.43	12.29 ± 1.63	-0.07 ± 1.41	.835	12.76 ± 2.70	0.97 ± 3.11	.346
Ascites	1.16 ± 0.38	1.26 ± 0.45	0.11 ± 0.46	.317	1.08 ± 0.29	0.08 ± 0.29	.317
HE	1.00 ± 0.00	1.00 ± 0.00	0	1.000	1.00 ± 0.00	0	1.000

Hb = hemoglobin, HE = hepatic encephalopathy, LY = lymphocyte, NEU = neutrocyte, NLR = neutrophil/lymphocyte ratio, PLT = platelet, PSE = partial splenic embolization, PT = prothrombin time, RBC = red blood cell, sd = standard deviation, TACE = transarterial chemoembolization, TB = total bilirubin, WBC = white blood cell. *P* is the comparison between 3 or 6 months after PSE+TACE and before PSE+TACE; 3–0: 3 months after operation compared with preoperative growth; 6–0: 6 months after operation compared with preoperative growth; *P* < .05 is defined as significant difference.

**Table 3:** Changes before and after TACE in the N-PSE group (means ± sd).

	Before TACE	3 months after TACE	3–0	*P*	6 months after TACE	6–0	*P*
WBC (×10^9^/L)	4.23 ± 1.42	4.23 ± 1.62	0.00 ± 1.46	.625	4.24 ± 1.13	-0.05 ± 1.04	.894
NEU (×10^9^/L)	2.58 ± 1.14	2.68 ± 1.31	0.11 ± 1.25	.994	2.77 ± 0.92	0.16 ± 0.87	.185
LY (×10^9^/L)	1.09 ± 0.31	0.96 ± 0.34	-0.13 ± 0.31	.015	0.91 ± 0.36	-0.22 ± 0.36	.007
NLR	2.62 ± 1.61	3.15 ± 2.15	0.53 ± 2.07	.034	3.48 ± 2.17	0.94 ± 2.10	.007
PLT (×10^9^/L)	99.41 ± 03.65	98.26 ± 41.80	-1.15 ± 33.04	.711	96.90 ± 35.07	-4.23 ± 34.73	.381
RBC (×10^12^/L)	4.18 ± 0.67	4.11 ± 0.68	-0.07 ± 0.48	.335	4.17 ± 0.72	-0.06 ± 0.58	.581
Hb, g/L	128.15 ± 20.41	127.10 ± 21.60	-1.05 ± 13.51	.667	125.47 ± 23.19	-4.03 ± 18.09	.232
Child-Pugh	5.74 ± 1.02	5.82 ± 1.36	0.77 ± 1.04	.770	5.63 ± 1.07	0.13 ± 1.01	.490
TB, μmol/L	29.52 ± 39.53	40.17 ± 90.31	10.66 ± 85.62	.733	26.71 ± 33.49	7.02 ± 33.01	.624
Albumin, g/L	38.78 ± 5.09	38.71 ± 6.39	-0.07 ± 4.78	.931	40.44 ± 5.55	0.80 ± 4.84	.373
PT, s	11.95 ± 1.32	12.19 ± 1.98	0.25 ± 1.92	.625	11.79 ± 1.66	0.04 ± 1.82	.643
Ascites	1.18 ± 0.39	1.26 ± 0.44	0.08 ± 0.27	.083	1.30 ± 0.47	0.17 ± 0.38	.025
HE	1.00 ± 0.00	1.00 ± 0.00	0	1.000	1.00 ± 0.00	0	1.000

Hb = hemoglobin, HE = hepatic encephalopathy, LY = lymphocyte, NEU = neutrocyte, NLR = neutrophil/lymphocyte ratio, N-PSE = non-partial splenic embolization, PLT = platelet, PT = prothrombin time, RBC = red blood cell, sd = standard deviation, TACE = transarterial chemoembolization, TB = total bilirubin, WBC = white blood cell. *P* is the comparison between 3 or 6 months after TACE and before TACE; 3–0: 3 months after operation compared with preoperative growth; 6–0: 6 months after operation compared with preoperative growth; *P* < .05 is defined as significant difference.

**Table 4:** Comparison of N-PSE and PSE (means ± sd).

	N-PSE 3–0	PSE 3–0	*P*	N-PSE 6–0	PSE 6–0	*P*
WBC (×10^9^/L)	-0.00 ± 1.46	1.75 ± 1.28	.000	-0.05 ± 1.04	2.61 ± 3.47	.001
NEU (×10^9^/L)	0.11 ± 0.20	1.04 ± 0.26	.005	0.16 ± 0.87	2.10 ± 3.18	.010
LY (×10^9^/L)	-0.13 ± 0.31	0.46 ± 0.47	.000	-0.22 ± 0.36	0.22 ± 0.42	.002
NLR	0.53 ± 2.07	0.35 ± 1.81	.358	0.94 ± 2.10	1.98 ± 3.13	.265
PLT (×10^9^/L)	-1.15 ± 33.04	58.05 ± 36.62	.000	-4.23 ± 34.73	45.00 ± 44.80	.000
RBC (×10^12^/L)	-0.07 ± 0.48	0.01 ± 0.47	.584	-0.06 ± 0.58	-0.24 ± 0.99	.749
Hb, g/L	-1.05 ± 13.51	1.95 ± 10.95	.404	-4.03 ± 18.09	-3.83 ± 29.93	.979
Child-Pugh	0.08 ± 1.04	0.37 ± 0.76	.097	0.13 ± 1.01	0.58 ± 1.44	.330
TB, μmol/L	10.66 ± 85.62	18.16 ± 75.47	.362	7.02 ± 33.01	14.02 ± 54.02	.867
Albumin, g/L	-0.067 ± 4.78	-1.24 ± 3.71	.353	0.80 ± 4.84	-1.70 ± 5.95	.165
PT, s	0.25 ± 1.92	-0.07 ± 1.41	.836	0.04 ± 1.82	0.97 ± 3.11	.365
Ascites	0.08 ± 0.27	0.11 ± 0.46	.725	0.17 ± 0.38	0.08 ± 0.29	.491
HE	0	0	1.000	0	0	1.000

Hb = hemoglobin, HE = hepatic encephalopathy, LY = lymphocyte, NEU = neutrocyte, NLR = neutrophil/lymphocyte ratio, N-PSE = non-partial splenic embolization, PLT = platelet, PSE = partial splenic embolization, PT = prothrombin time, RBC = red blood cell, sd = standard deviation, TB = total bilirubin, WBC = white blood cell. 3–0: 3 months after operation compared with preoperative growth; 6–0: 6 months after operation compared with preoperative growth; *P* is the comparison between groups; *P* < .05 is defined as a significant difference.

### 3.2 Survival analysis and prognostic factors for survival

**Figure 4:**
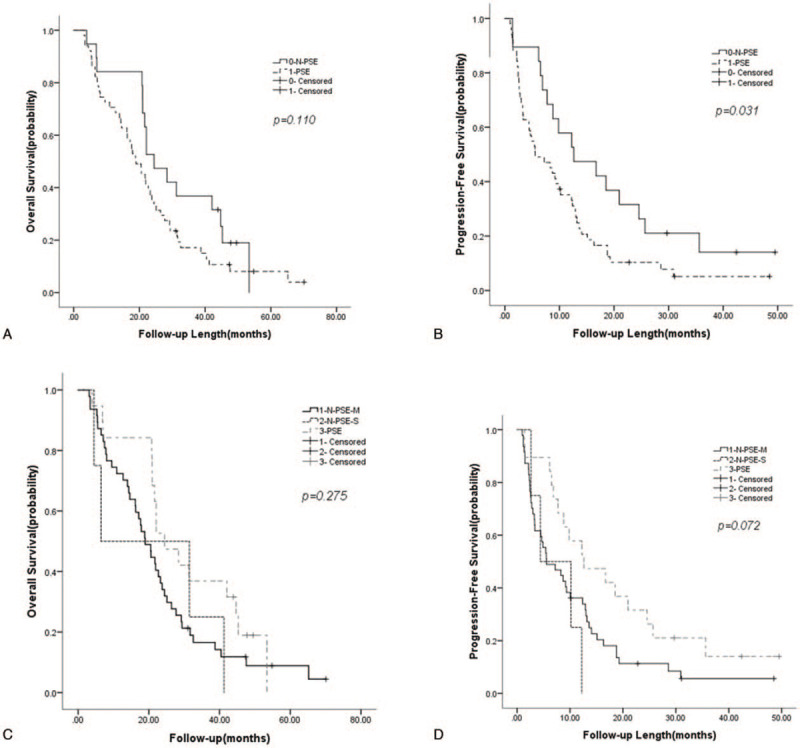
Kaplan–Meier estimates of overall survival and progression-free survival. (A) OS survival curve between 2 groups. (B) PFS survival curve between 2 groups. (C) OS survival curve between 3 groups. (D) PFS survival curve between 3 groups. OS = overall survival, PFS = progression-free survival.

As shown in Table 5, according to the follow-up results, the median OS in the PSE group was 24.47 ± 3.68 (months), and the PFS was 12.63 ± 4.98 (months). Regardless of OS or PFS, the PSE group was better than the N-PSE group and its subgroups. The difference in PFS between the N-PSE group and the PSE group was statistically significant (5.53 ± 2.06 vs 12.63 ± 4.98, *P* < .05) (Fig. 4). Further analysis of PFS showed that both the PSE group and the N-PSE-M group first had intrahepatic in situ progression. In contrast, the N-PSE-S group first had extrahepatic progression. Also, the time of extrahepatic progression in the N-PSE group was significantly earlier than that in the PSE group (12.67 ± 4.97 vs 22.10 ± 0.84, *P* < .05). In the group with severe hypersplenism, TACE without PSE resulted in the worst prognosis, and PFS and OS were worse than in the other 2 groups, which suggested that PSE in severe hypersplenism may improve PFS and OS.

**Table 5:** Prognosis of each group (median + sd, months).

		N-PSE, mo				
	N-PSE, mo	N-PSE-M	N-PSE-S	PSE, mo	P1	P2
OS	18.97 ± 2.35	18.97 ± 2.01	6.50 ± 13.50	24.47 ± 3.68	0.110	0.275
PFS	5.53 ± 2.06	5.53 ± 2.31	4.40 ± 3.77	12.63 ± 4.98	0.031	0.072
Intrahepatic in situ progression	9.53 ± 2.08	9.53 ± 2.06	6.50 ± 3.90	18.53 ± 6.34	0.056	0.148
Intrahepatic neoplastic metastasis	10.17 ± 2.19	12,77 ± 2.86	6.50 ± 2.89	22.30 ± 6.94	0.051	0.149
Extrahepatic progression	12.67 ± 4.97	12.67 ± 3.65	4.40 ± 9.85	22.10 ± 0.84	0.028	0.088

N-PSE = non-partial splenic embolization, OS = overall survival, PFS = progression-free survival, PSE = partial splenic embolization, sd = standard deviation. P1 comparison between large groups; P2 comparison between subgroups; *P* < .05 is defined as a significant difference.

Further analysis of the influencing factors of OS suggested that univariate BCLC stage C and female sex were risk factors for poor prognosis (*P* < .05). Multivariate analysis suggested that female sex and liver cirrhosis were independent risk factors for poor prognosis (*P* < .05), while PSE might be a protective predictor (*P* = .079) (Table 6).

**Table 6:** Univariate and multivariate analysis affecting OS.

	Univariable analysis			Multivariable analysis		
Parameter	Hazard ratio	95%CI	*P*-value	Hazard ratio	95%CI	*P*-value
N-PSE/PSE	0.629	0.354 1.116	.113	0.563	0.296 1.069	.079
Gender Male/Female	3.556	1.481 8.539	.005	4.412	1.745 11.153	.002
Age Elderly/middle-aged	1.200	0.720 2.000	.484			
Hepatitis	1.623	0.688 3.830	.269			
Liver cirrhosis	0.602	0.345 1.052	.075	0.554	0.315 0.973	.040
Stage C/ B	0.506	0.271 0.943	.032	0.684	0.298 1.572	.371
>10 cm/<10 cm	0.840	0.507 1.389	.496			
Pseudocapsule	0.632	0.345 1.156	.136	0.524	0.270 1.017	.056
Evagination	1.501	0.713 3.160	.285			
AFP >400/<400	1.229	0.743 2.033	.421			
Location Left lobe/Right lobe	0.726	0.392 1.346	.310			
Portal vein tumor thrombus	0.630	0.380 1.045	.074	0.834	0.433 1.606	.586
Arteriovenous fistula	0.592	0.319 1.099	.097	0.682	0.350 1.328	.260
Extrahepatic metastasis before initial treatment	0.756	0.390 1.465	.407			
Other treatments	0.757	0.432 1.328	.332			

AFP = alpha-fetoprotein, CI = confidence interval, N-PSE = non-partial splenic embolization, OS = overall survival, PSE = partial splenic embolization. *P* < .05 is defined as a significant difference.

## 4 Discussion

The splenomegaly is most commonly caused by blood disease, liver disease, and infections, where liver disease accounts for 23%.^[10,11]^ More than 85% of HCC patients have liver cirrhosis,^[4]^ so HCC with splenomegaly and hypersplenism are prevalent. Hematological abnormalities caused by hypersplenism may limit HCC treatment options and delay the treatment implementation.^[12,13]^ People with severe hypersplenism have a higher risk of varicose vein rupture and bleeding, and even death.^[14,15]^ Still, the use of β-blockers or antibiotics for medical treatment of severe hypersplenism has no definite benefit,^[15]^ making invasive treatment an important option for hypersplenism.

The incidence of complications following splenectomy in hospitalized patients with splenomegaly in the United States has been reported to be 6% to 22%,^[10]^ with a mean blood loss of 354 mL and an incidence of portal thrombosis of 10%.^[8]^ Sugawara et al^[16]^ reported that after undergoing simultaneous or staged splenectomy and liver tumor resection, the incidence of biloma and ascites was about 50% in patients with HCC and hypersplenism. The incidence of vicious infection after splenectomy was 3.2%,^[17]^ and the mortality rate was as high as 50%.^[18]^ It was particularly important to avoid susceptibility to infection due to the absence of the spleen or significantly reduced splenic function in those with concomitant HCC, where the organism was in a tumor-tolerant state.^[12]^

Compared with the poor efficacy of medical therapy and the trauma and high complication rate of splenectomy, PSE has been increasingly recommended for the treatment of hypersplenism. During PSE, adequate embolization of the spleen through arterial injection of the embolic agent may reduce the volume of the spleen and preserve part of the spleen function, thus ensuring the smooth treatment of HCC.^[12]^ Besides, the tolerance of simultaneous TACE+PSE was not worse than TACE and delayed PSE.^[5]^ For patients with HCC and hypersplenism, TACE combined with PSE was more suitable than splenectomy.^[12]^

The results of this study suggested that regardless of OS or PFS, the PSE group had the best results, and the severe hypersplenism group had the worst, which suggested that TACE combined with PSE might improve PFS and OS in patients with severe hypersplenism. Studies on the effects of splenic embolism on liver function did not yield positive results, that is, there was no significant difference in liver function scores before and after PSE. Nevertheless, a subgroup analysis showed that the ascites in the N-PSE group significantly increased 6 months after TACE, thus suggesting that PSE may help slow down the increase in ascites. This may be due to the following: PSE caused a general rebound of blood cells, and the elevation of blood cells was positively correlated with the rate of splenic embolism.^[13]^ The degree of embolization was preferably 50% to 70%.^[7]^ When the embolization area is <50%, the increase in blood cells is not obvious.^[8]^ When the embolization area was >70%, complications increased.^[7]^ PSE removed the limitations of low blood cells on TACE, ablation, and other treatment methods, so patients could be treated as scheduled.^[7]^ The recovery of WBCs and PLTs avoids the common fatal complications caused by low blood cells such as infection and bleeding. The improvement in anemia led to the restored function of various organs and increased the sensitivity of tumor cells. Furthermore, PSE can improve blood cell composition and improve immune function. After PSE, total T cells, total helper T-cells (CD4), total suppressor T-cells (CD8), and natural killer cells were all increased.^[19]^

Chu et al^[18]^ studied the function of the remaining spleen after partial splenic embolization. The results showed that in the remaining spleen, T lymphocytes were mainly gathered in the peripheral lymphatic tissue sheath of the splenic corpuscle. In the red pulp, macrophages were evenly distributed in the spleen cord. The remaining spleen splenic corpuscles per unit area of T lymphocytes and red pulp mid-marginal macrophages were significantly increased. The number of peripheral blood PLTs and WBCs, the plasma ratio of CD3+ T cells, CD4+ T cells, and CD8+ T cells also significantly increased. Yet, the macrophage colony-stimulating factor (M-CSF) and granulocyte-macrophage colony-stimulating factor (GM-CSF) in plasma were significantly reduced. The increase in the proportion of CD4+ T cells and natural killer cells can improve host immunity and promote anti-tumor effects.^[19,20]^

M-CSF can induce monocytes to produce more T-helper 2 (Th2) cytokines and less T-helper 1 (Th1) cytokines. It has been confirmed that in HCC tissues, the decrease in Th1 cytokine expression and the increase in Th2 cytokine expression are associated with vascular invasion and metastasis recurrence.^[21]^ M-CSF could also cause macrophages to be of M2 type,^[22]^ which produce cytokines and growth factors that inhibit immune function and promote tumor progression. They also produce growth factors related to tissue proliferation and angiogenesis that promote tumor recurrence and metastasis, such as interleukin-10 (IL-10), transforming growth factor -beta, epidermal growth factor, insulin-like growth factor, vascular endothelial growth factor, PLT-derived growth factor and similar.^[21]^ GM-CSF was the determinant of malignant tumor cells-mediated neutrophil production of hepatocyte growth factor. Tumor neutrophils could activate and enhance the metastasis of malignant tumor cells through the hepatocyte growth factor /c-Met interaction.^[23]^ The reduction of M-CSF and GM-CSF after PSE could reduce their negative regulatory effect on the immune system and enhance the anti-tumor effect. In this study, patients with severe hypersplenism who did not undergo PSE had the earliest extrahepatic progression, and PFS was significantly prolonged in the PSE group compared with the N-PSE group, which suggested that PSE may improve the immunity in patients with HCC and hypersplenism, which is beneficial to tumor control.

Finally, some researchers believe that PSE can improve liver function. Lee et al^[24]^ reported that liver function improved from grade B to grade A in 3 out of 4 cases at 2 months after PSE. Ishikawa et al^[12]^ also showed that 18 patients with HCC and thrombocytopenia had significant improvement in liver function after simultaneous TACE and PSE compared with TACE alone (*P* < .05). In a study conducted by Toru Ishikawa that included 101 patients, 53 patients underwent TACE and PSE at the same time, and 48 patients underwent TACE alone. The former's Child-Pugh liver function improved after a brief deterioration lasting for 2 weeks after surgery, while the latter's liver function continued to deteriorate.^[12]^ The PSE improvement in liver function was mainly based on a decrease in portal pressure and a slowing of the fibrosis process.

PSE reduces the blood flow through the splenic artery into the portal artery, resulting in a 30% to 50% reduction in portal pressure^[5]^ and a reduction in ascites. Reduced portal pressure also slows the progression of liver cirrhosis and increases liver cell regeneration.^[25,26]^

After the liver injury, Th2-dominant splenic T lymphocytes migrate into the liver and develop toward Th2-dominance by changing the balance of Th1/Th2. Th2 cytokines such as IL-4 and IL-13 promote hepatic stellate cell activation and accelerate the process of liver fibrosis. PSE can reduce the absolute number of Th2 lymphocytes, restore the Th1/Th2 imbalance, to the advantage of Th1, thereby inhibiting liver fibrosis.^[26,27]^ Also, thrombocytosis after PSE can also inhibit liver fibrosis.^[28]^ Improved liver function can enhance liver protein synthesis and reduce the severity of hepatic encephalopathy.^[7]^

There were some deficiencies in this study, such as, some cases were clinically diagnosed, without pathological classification, and these might produce selection bias. PSE was not treated at the first visit and TACE treatments might aggravate hypersplenism, which might affect the choice of PSE group patients to a certain extent. In addition, the number of patients with severe hypersplenism without PSE was less, which might affect the statistical results.

In conclusion, the combination of TACE and PSE could effectively increase the peripheral blood cell level in patients with HCC and severe hypersplenism. Since there was no obvious liver damage compared with TACE alone, HCC could be used as regular and standardized treatment. TACE combined with PSE could significantly increase PFS, while further studies are necessary to establish if it is possible to increase OS. In patients with HCC and severe hypersplenism, TACE treatment should be actively combined with PSE therapy.

## Acknowledgments

The authors thank the doctors, nurses, technicians, and the second ward of the Interventional Department of Shandong Cancer Hospital and Institute in Shandong Province, China, for their assistance with and support for this project.

## Author contributions

**Conceptualization:** Jibing Liu.

**Data curation:** Zhijuan Wu, Jianxin Zhang.

**Data Interpretation:** Jibing Liu, Huiyong Wu, Lin Zhang.

**Formal analysis:** Jianxin Zhang.

**Investigation:** Yinfa Xie.

**Literature Search:** Jianxin Zhang, Xu Chang.

**Manuscript Preparation:** Zhijuan Wu, Peng Sun, Lin Zhang.

**Project administration:** Peng Sun.

**Resources:** Huiyong Wu.

**Software:** Xu Chang.

**Statistical Analysis:** Lin Zhang, Fengyong Liu.

**Study Design:** Jibing Liu, Lin Zhang, Yinfa Xie

**Visualization:** Fengyong Liu.

**Writing – review & editing:** Lin Zhang.
